# Knowledge, Attitudes, and Biosecurity Practices Regarding African Swine Fever Among Small-Scale Pig Farmers in the Lao People’s Democratic Republic and Cambodia

**DOI:** 10.3390/v18010034

**Published:** 2025-12-24

**Authors:** Véronique Renault, Ariane Masson, Paeng Xaphokame, Outhen Phommasack, Borin Sear, Samnang Ven, Claude Saegerman

**Affiliations:** 1 Research Unit of Epidemiology and Risk Analysis Applied to Veterinary Sciences (UREAR-ULiège), Fundamental and Applied Research for Animal and Health (FARAH) Center, Faculty of Veterinary Medicine, University of Liège, 4000 Liège, Belgium; vrenault@uliege.be; 2 Independent Veterinary Practitioner, 49700 Doué La Fontaine, France; 3 Agronomes et Vétérinaires Sans Frontières, Vientiane 01000, Laos; 4 Agronomes et Vétérinaires Sans Frontières, Phnom Pen 12000, Cambodias.ven@avsf.org (S.V.)

**Keywords:** ASF, KAP survey, Lao PDR, Cambodia, biosecurity, small-scale farming, swine

## Abstract

African swine fever (ASF) is a transboundary viral disease that has heavily impacted Southeast Asia since its introduction in 2019. Smallholder pig production systems in Cambodia and the Lao People’s Democratic Republic (the Lao PDR), characterized by low biosecurity, free-ranging practices, and limited veterinary oversight, remain particularly vulnerable. To assess farmers’ awareness and practices regarding ASF, a knowledge, attitudes, and practices (KAP) survey was implemented between March and September 2023 by Agronomes et Vétérinaires Sans Frontières within the framework of the Biosecurity in Pig Farming (BIG) project. A total of 471 pig farmers, including 56% women, were interviewed across eight provinces using a standardized questionnaire (188 in Cambodia and 283 in the Lao PDR). Results showed that ASF awareness was generally high (92% in Cambodia, 66% in the Lao PDR), yet 15% of Cambodian and 30% of Lao respondents expressed doubts about the presence of ASF in their country. While recognition of ASF symptoms was moderate and positively correlated with farmers’ perceived capacity to identify the disease, knowledge of transmission pathways was low and often misaligned with perceptions. Airborne transmission was frequently cited as a risk, and the risks related to visitors and fomites were underestimated by more than 50% of the farmers. Implementation of biosecurity measures (BSM) was limited, with mean scores of 43% in Cambodia and 27% in the Lao PDR. Risky practices such as swill feeding, free-ranging, sharing of boars, traders, and inadequate carcass disposal remained widespread. Statistical analysis identified education level, herd size, knowledge, perceived risks, and perceived benefits of BSM as the main determinants of biosecurity implementation. Farmers with larger herds or stronger commercial orientation demonstrated higher biosecurity adoption, while misconceptions and knowledge gaps remained frequent among smallholder farmers. Strengthening awareness, promoting low-cost and feasible biosecurity practices, and integrating farmer-centred approaches are essential for reducing ASF transmission risks and improving the resilience of smallholder pig production systems in the region.

## 1. Introduction

African swine fever (ASF) is a highly contagious viral disease affecting both domestic and wild pigs. It is caused by the African swine fever virus (ASFV), a large double-stranded DNA virus belonging to the family *Asfarviridae* [1]. The disease is characterized by extremely high morbidity and mortality rates, which can reach 100% in susceptible populations. Consequently, ASF is considered one of the most significant transboundary animal diseases worldwide, with severe socioeconomic and food security implications [2,3].

The first reported outbreak of ASF in Southeast Asia occurred in Vietnam in early 2019, followed shortly thereafter by cases in Cambodia, China, and the Lao People’s Democratic Republic [4]. Specifically in Cambodia and the Lao PDR, the first outbreaks were reported in March and June 2019, respectively, followed by rapid dissemination throughout both countries [5,6]. Between March and July 2019 in Cambodia, continuous outbreaks were reported in five provinces, Rattanakiri, Tboung Khmum, Svay Rieng, Takeo, and Kandal, resulting in the mortality or culling of 5000 pigs [7]. In the Lao PDR, a report from July 2019 detailed a total of 23 villages affected in 6 provinces with a total of 3163 pigs either dead or culled in a pig population of 4898 pigs [8]. From June to December 2019, 150 outbreaks were investigated across all provinces [9]. Weak national border biosecurity and limited surveillance capacity contributed to the virus’s introduction, establishment, and spread in backyard and semi-commercial production systems. Since 2019, outbreaks have been repeatedly confirmed in both countries, with a spillover of the disease into wild boar populations in the Lao PDR and Vietnam [10].

Pig production in this region is dominated by smallholder systems, in which pigs play a critical role in household food security, cultural practices, and rural livelihoods [4,9]. However, these systems are typically characterized by low biosecurity standards, unrestricted animal movement, and limited veterinary oversight [11]. In such contexts, ASF control is particularly challenging in the absence of an effective and safe vaccine. Although one commercially available vaccine is currently used in selected countries such as Vietnam and the Philippines, it has not been authorized in other countries, including the Lao PDR and Cambodia, due to concerns regarding the dissemination of vaccine-derived variants [12]. Therefore, biosecurity remains the only approach for prevention and control of ASF in the Lao PDR [1], and several training and awareness-raising campaigns have been organized throughout the country to encourage the adoption of strengthened biosecurity measures by the pig farmers [8]. Nevertheless, awareness and implementation of biosecurity measures remain limited among small-scale farmers, where free-ranging pig husbandry is still widely practiced [13].

It has been demonstrated that the farmers’ decision to implement good practices does not rely solely on technical awareness but also on other mental constructs such as the perceived susceptibility and impact of the risk to be addressed, the perceived benefits of the measures, the beliefs and attitudes of the farmers, and contextual constraints [14,15,16,17,18,19]. Theory-driven interventions are therefore highly recommended to increase the effectiveness of the awareness-raising campaigns and/or training as they allow for clarifying the target groups and the determining factors of behaviour change, which might vary from one risk to another [14,18,20,21].

A knowledge, attitudes, and practices (KAP) survey was conducted between March and September 2023 within the framework of the Biosecurity in Pig Farming (BIG) project, funded by the French Ministry of Foreign Affairs. The survey was implemented by Agronomes et Vétérinaires Sans Frontières (AVSF) in collaboration with the French National School of Veterinary Services (ENSV-FVI) and the French Agricultural Research Centre for International Development (CIRAD). The survey covered eight provinces, five in Cambodia and three in the Lao PDR. Its objectives were (i) to assess farmers’ knowledge and perceptions of ASF, and (ii) to identify the determining factors of biosecurity measures implementation. The findings of this survey should allow the veterinary services to identify knowledge gaps and better design the communication tools based on targeted awareness strategies.

## 2. Materials and Methods

### 2.1. Study Sites

**Figure 1:**
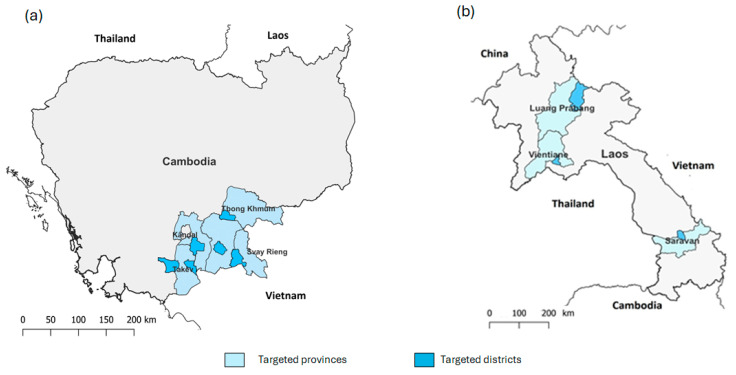
Map of survey areas in (**a**) Cambodia and (**b**) the Lao PDR.

In Cambodia, six districts in five provinces were selected for the study: Svay Rieng, Tboung Khum, Takeo, Kandal, and Prey Veng provinces (Figure 1a). These districts were selected based on the recommendation from the animal health authority institution and the data obtained and reported by the technical services and the office of animal health and production in each province of the study. These provinces are in the southeastern part of Cambodia, where the first outbreaks of African swine fever were officially reported in 2019. These provinces share borders with Vietnam in the South and represent the main land corridor with Vietnam, supporting trade, travel, and cultural exchanges between the 2 countries. The topography of all five provinces is dominated by flat lowland plains, shaped by the Mekong and Bassac river systems, making them fertile zones for rice and other crops. Takeo and Tboung Khmum stand out slightly, with Takeo hosting small limestone hills and Tboung Khmum having gentle uplands. In contrast, Kandal, Prey Veng, and Svay Rieng are almost entirely alluvial plains prone to seasonal flooding, forming Cambodia’s agricultural heartland along the Vietnam border. All provinces are low-altitude areas, mostly under 40 m, except for isolated hills in Takeo. Temperatures are consistently hot and humid, with minor seasonal cooling, and the altitude difference is too small to affect climate significantly (except for slightly cooler nights in Takeo’s hill areas). Smallholder farming households expanded livestock production, which now accounts for 11.7 percent of agricultural gross domestic product [22]. Livestock production grows at 5.5 percent annually, driven by poultry and pigs [23,24]. In backyard farming, pigs are mostly raised for live sale, self-consumption, and breeding purposes. In 2023, the pig population held by households varied across the five provinces. Prey Veng recorded 30,386 pigs, Svay Rieng had 80,191 pigs, Takeo had 240,049 pigs, Tboung Khmum recorded 21,245 pigs, and Kandal recorded 20,161 pigs [25]. Within each district, one to four villages were identified by district veterinary services based on the number of active pig farmers. A total of 188 farmers (mean: 12.5 ± 6 farmers per village) were interviewed face-to-face.

In the Lao PDR, three provinces were selected to reflect different pig production contexts and ASF epidemiology: Saravan (the site of the first outbreak in the country), Luang Prabang (where spillover into wild pigs was documented), and Vientiane Capital (with a higher proportion of commercial farms). Saravan is located on the Bolaven plateau and borders Vietnam to the east and Thailand to the west. The topography includes plains and mountainous regions, as well as two protected areas. It sits at an altitude of 400–2100 metres with an average temperature of 27.3 °C. The percentage of farming households is 55%, among which 52.39% have a sole commercial purpose, and 47.61% are subsistence and commercial farming. Luang Prabang province is a mountainous area with the uplands being quite isolated, especially in the rainy season. The pig production relies mainly on small-scale/backyard farming with a free-ranging system. The survey area borders Nam-Et-Phouylouy National Park. Vientiane province is in the middle of the country and includes a higher proportion of commercial farms. Pig and population movements are much higher, and pig raising is mainly commercial. Within each province, one district was arbitrarily selected (Figure 1b). In each district, seven to eight villages were randomly chosen. A total of 283 farmers (mean: 13 ± 4 farmers per village) were interviewed.

In both countries, farmers were recruited voluntarily after holding a village information session. Eligibility criteria included being over 18 years old and actively raising a minimum of one pig. Commercial farms raising pigs under an intensive production system were excluded from the survey.

### 2.2. Questionnaire Survey

The questionnaire was designed using KoboToolbox (version 2023), pre-tested with field staff, and subsequently finalized for deployment. It consisted of 126 closed questions and sub-questions grouped into five sections: (i) respondent profile, (ii) farm characteristics, (iii) knowledge, (iv) attitudes/perceptions, and (v) practices. Initially drafted in English, the questionnaire was translated into Khmer and Lao. Data collectors were trained in the use of the KoboCollect (version 2023) application and equipped with tablets for digital data collection.

### 2.3. Data Extraction and Scores

Data were extracted using Microsoft Excel (version 2025) before descriptive analysis. Scores were developed to quantify three mental constructs: (i) ASF knowledge, (ii) risk perception of ASF, and (iii) perceived benefits of the biosecurity measures (BSM). Biosecurity scores were also calculated for each farm to be used as dependant variable. Knowledge scores were based on recognition of ASF transmission pathways and clinical signs, with higher weight given to highly-specific signs such as fever, sudden death, cutaneous hemorrhages (abdomen, ears, tail, or limbs), and haemorrhagic excretions (feces, urine, nasal/ocular discharge). Risk perception was assessed via structured questions (Table 1), as well as the perceived benefits of BSM, calculated based on the perceived benefits of each BSM. The BSM implementation was quantified based on the presence or absence of 27 practices (Appendix A). The four scores were normalized to percentages (0–100):

Knowledge score (KS) = (K1 + K2 + K3)/3 × 100(1)

where K1 = 0 or 1 if the answer is no or yes to the following question “Have you ever heard about ASF?”; K2 is the sum of the clinical signs of ASF that the farmer associated correctly to ASF (four most important clinical signs received a score of two in place of one, see Appendix A for details) divided by the maximum number of points possible; K3 is the sum of the correct spread pathways through which pigs can be infected by ASF, divided by the maximum number of points possible.

Risk perception score (RP) = (RP1 × RP2 + RP3 × RP4 × RP5 + RP6)/10 × 100(2)

where RP1 = 0 or 1 if the answer is no or yes to the following question “Have you ever experienced African swine fever outbreak on your farm?”; RP2 = 0 or 1 if the answer is no or yes to the following question “Did any of your pigs died from ASF (after being sick or killed by local authorities) during outbreak?”; RP3 = 0 or 1 if the answer is no or yes to the following question “Do you know anybody who has been affected by ASF?”; RP4 = 1 or 2 if the answer is another pig farmer/relative or a friend to the following question “Who do you know and has been affected by ASF?”; RP5 = 1 or 2 if the answer is another village or the same village to the following question “Where these persons affected by ASF keeping the pigs?”; RP6 = the sum of correct assumptions (for details see Appendix A) divided by four.

Perceived importance measures (PI) = Sum PCM/25 × 100(3)

where PCM is the number of prevention and control measures considered as efficient/important by the farmer, and 25 is the maximum number of points possible.

Biosecurity score (BSc): = (P1 × P1.1 × P1.2 + P2 + P3 + P4 + P5 + P6)/41 × 100(4)

where P1, P1.1 and P1.2 are, respectively, questions about the penning, pen flooring and pen walls; P2 is question about the feeding of pigs; P3 is question about the action in case of ASF clinical signs in pigs; P4 is question about the action following the discovery of an ASF outbreak in the farm/village; P5 is question about the carcass disposal; P6 is question about the implementation of biosecurity measures in the farm (for more details, see Appendix A); and 41 is the maximum number of points possible.

### 2.4. Statistical Analyses

Normality of the dependent variable (biosecurity score) was assessed using histograms and tested using the Shapiro–Wilk test (Stata SE 14.2, College Station, TX, USA).

To ascertain whether the ranking of biosecurity score (BS) at the farm level was influenced by the parameters included in the calculation of the BS, a sensitivity analysis was performed using a comparison of the ranking of farms without considering each parameter, with the ranking of farms with consideration of all parameters as reference. The difference between the above ranking of farm (all parameters as reference) and each other ranking (in each case, one parameter was missing) was tested using the Pearson coefficient of correlation test [26]. When the *p*-value was less than 0.05, the correlation between the two rankings tested was considered significant, and the pair comparison with the lower coefficient of correlation (r) indicated the importance of this missing parameter in the BS.

The rank biserial correlation coefficient (rrb) was used to test the correlation when one of the variables is dichotomous, and the other variable is metric but not parametric [26]. The rrb was used to evaluate associations between perceived and actual knowledge regarding ASF transmission pathways and clinical signs (Stata SE 14.2, College Station, TX, USA). Regression tree analysis (RTA) was applied to identify the cut-off that separates the bimodal distribution of biosecurity score observed in Lao (Salford Predictive Modeler, Salford Systems, San Diego, CA, USA). Classification tree analysis (CTA) was then applied to identify explanatory variables influencing biosecurity scores either directly or indirectly (Salford Predictive Modeler, Salford Systems, San Diego, CA, USA). Due to differences in sampling methodologies, cross-country comparisons were not performed; data were analyzed separately for each country. A classification and regression tree analysis (CART) is a discrimination method based on the construction of a binary decision tree. The goal is to construct subgroups of a population that are as homogeneous as possible for a given characteristic (variable to be explained) (for more details, see, e.g., ref. [27]).

## 3. Results

### 3.1. Descriptive Analysis

A total of 471 pig farmers (188 in Cambodia and 283 in the Lao PDR) were interviewed, of whom 56% were women (Table 2). The majority were raising pigs for more than five years, and pig farming represented their main or secondary source of household income (72% in Cambodia; 57% in the Lao PDR). Awareness of ASF was high in Cambodia (92%) but lower in the Lao PDR (66%). More than half of the respondents directly experienced an ASF outbreak (Table 3).

Notably, 15% of Cambodian and 30% of Lao farmers expressed doubts about the existence of ASF in their country. Nevertheless, most farmers considered ASF to be both important and frequent in their country, and more than 50% acknowledged the need for specific preventive measures in the absence of vaccination or prophylactic treatments (Table 4).

**Figure 2:**
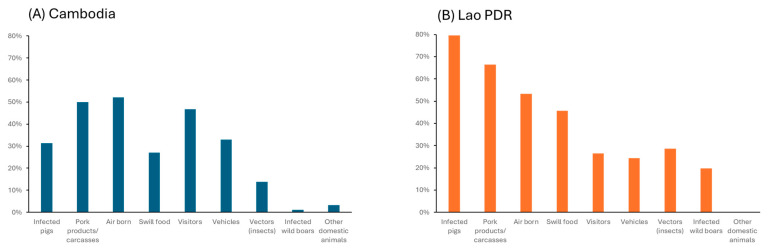
Perceived transmission pathways listed by the farmers in (**A**) Cambodia and (**B**) the Lao PDR.

Knowledge of ASF transmission was variable. More than 50% of farmers in both countries identified airborne transmission, contact with infected carcasses, and contaminated pork products as possible pathways (Figure 2). Thirty-two percent of Cambodian farmers identified contact with infected pigs, against 79.5% in the Lao PDR. Visitors were considered as a potential component of the risk pathway by 47% of Cambodian farmers, but by only 27% in the Lao PDR. Feeding contaminated swill was recognized as a risk by 27% and 46% of farmers in Cambodia and the Lao PDR, respectively.

**Figure 3:**
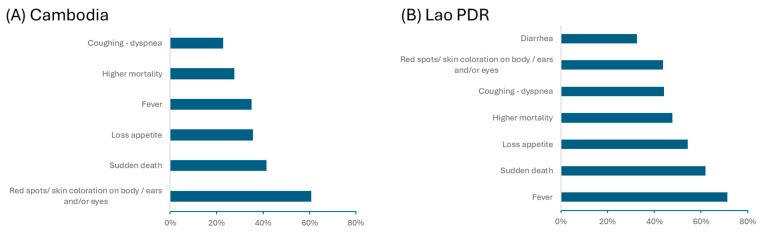
List of clinical signs most frequently reported by the farmers in (**A**) Cambodia and (**B**) the Lao PDR.

The six most frequently cited ASF symptoms were fever, sudden death, high mortality rates, loss of appetite, respiratory signs, and red skin lesions (Figure 3).

**Figure 4:**
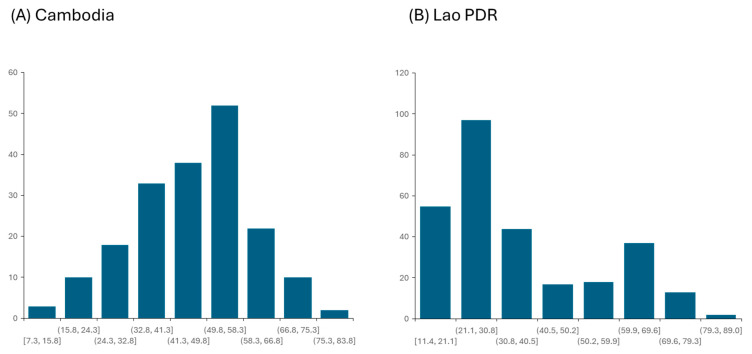
Distribution of the biosecurity scores in (**A**) Cambodia and (**B**) the Lao PDR.

**Figure 5:**
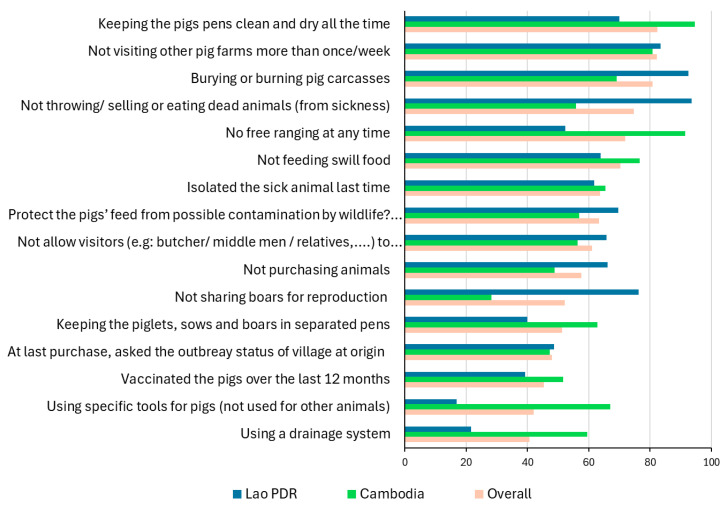
Biosecurity measures are most implemented in Cambodia, the Lao PDR, and overall.

BSM implementation was limited in both countries. In Cambodia, overall implementation scores ranged from 7% to 79% (mean: 44.3% ± 6), while in the Lao PDR, they ranged from 11% to 82% (mean: 27% ± 16) (Figure 4). Several high-risk practices, such as free ranging, swill feeding, sharing boars for mating, and carcass mismanagement, were still frequently reported in both countries (Table 5). Measures with implementation levels above 40% are shown in Figure 5.

### 3.2. Statistical Analysis

The result of the sensitivity analysis indicated that, irrespective of the parameter excluded, excluding some parameters had significant effects on the ranking of BS by farm compared to the reference (all parameters included). The top-three parameters who contributed more to the calculation of BS (in decreasing g order) were P1.2 (the pen walls, either made of wooden fence, steel fences or cemented walls) with r = 0.879; P6 (implementation of 26 BSM in the farm (see Appendix A)) with r = 0.901; and P1 (the penning system: free ranging, part-time penning and full-time penning) with r = 0.934.

The correlation between the knowledge scores for the ASF symptoms and transmission pathways and the perceived knowledge of the farmers on these two topics was assessed. Although a significant correlation was found for the perceived knowledge and actual knowledge of the symptoms (rank biserial correlation coefficient: rrb = 0.2153, *p*-value = 0.0001), no correlation was found for the transmission pathways (rank biserial correlation coefficient: rrb = 0.04 and *p*-value = 0.39).

As shown in Figure 4 and confirmed by a Shapiro–Wilk test, the biosecurity score follows a normal distribution in Cambodia (*p*-value = 0.40) but presents a bimodal distribution in the Lao PDR (*p*-value < 0.00001). The cut-off value was determined as 45 by visual observation of the bimodal distribution of biosecurity score (BS) and confirmed using a regression tree analysis (RTA). A classification tree analysis (CTA) was then performed to compare the 2 groups and identify possible explanatory variables.

**Figure 6:**
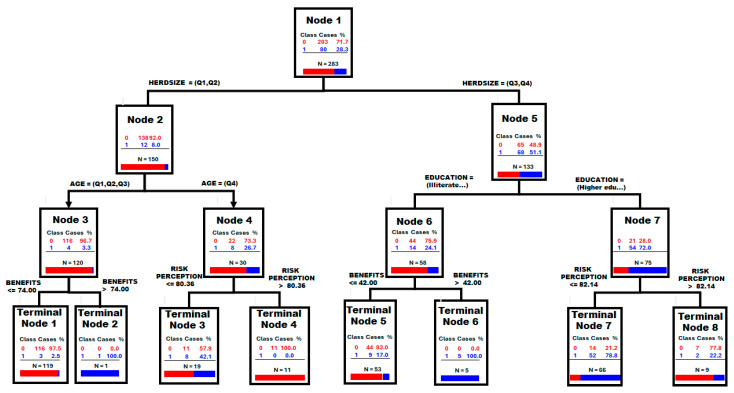
Classification tree analysis using the Lao PDR farms regrouped in two categories based on their biosecurity score being over or below 45 as dependant variable. Legend: Horizontal bars: in red, non cases and in blue, cases. The discrimination power of the classification tree analyzed (CTA) was assessed using the area under the receiver operating characteristic curve (AUC-ROC) with a theoretical maximum value of one; AUC-ROC of the CTA = 0.95 and 0.79 for the learning data set that permits the construction of the tree and for the testing data set that permits the testing the predictability of the tree, respectively.

According to the CTA, four factors exhibited an importance exceeding 80% in explaining the BS score: the education level, the perceived benefits, the herd size, and the risk perceptions over 80% (Table 6). The initial nodes of the classification tree are shown in Figure 6. The first node is defined by the herd size, using the median as the cut-off value. Subsequent nodes are determined by the farmers’ age in the category with the lowest BS score and the education level in the group with the highest score.

**Figure 7:**
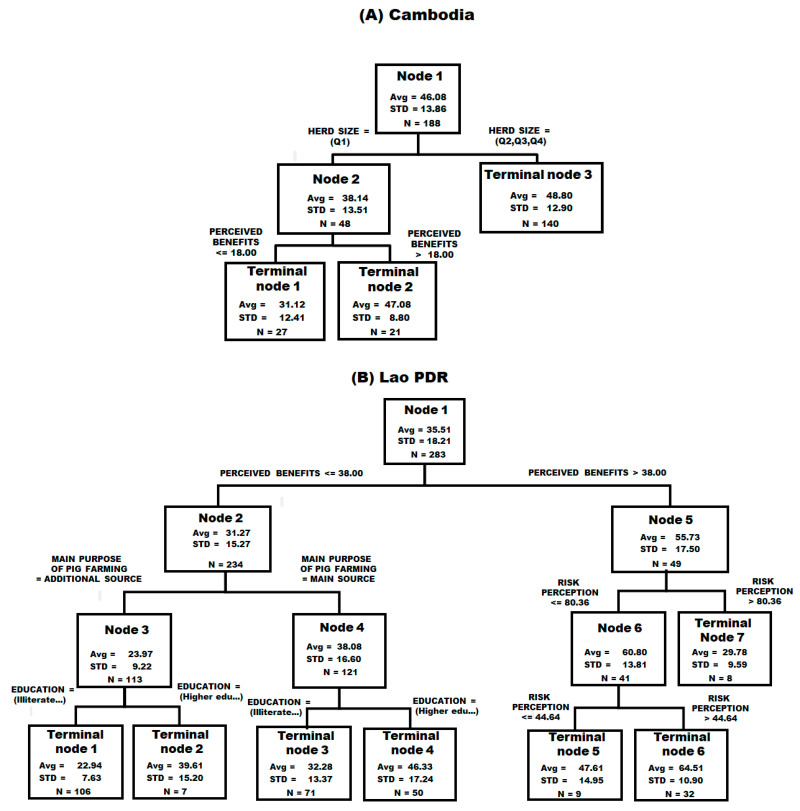
Regression tree analysis for (**A**) Cambodia and (**B**) the Lao PDR using biosecurity scores as the dependent variable.

Based on the RTA, several explanatory variables were identified as having a substantial importance on the BS score (Table 7). In Cambodia, the biosecurity score seems mainly predicted by the herd size and the perceived benefits, as confirmed by the RTA (Figure 7A). The perceived benefits are positively influenced by a bigger herd size, the importance of the pigs for the household livelihoods, and the commercial purpose of the pig farming. In the Lao PDR, the education level, as well as the three mental constructs, seem to have the biggest influence on the BS score (Table 7 and Figure 7B). The ASF knowledge is influenced mainly by the education level and the age of the farmer. The perceived risk of ASF as well as the perceived benefits of the BSM are mainly influenced by the herd size, the education, and the importance of pig farming for the farmers’ livelihoods.

## 4. Discussion

Knowing the practices and beliefs of small-scale pig farmers is key to understanding the challenges and constraints to behavioural change and properly supporting communication strategies and activities aiming at strengthening the BSM among small-scale farmers. A few studies in the Lao PDR described these practices [5,28,29,30]. Nevertheless, this survey sample size (188 in Cambodia and 283 in the Lao PDR) is larger than the previous studies in the region. In both countries, provinces and districts were selected arbitrarily but are considered representative of the diversity of contexts. In each of the districts, villages were randomly chosen in the Lao PDR, and in Cambodia, the district veterinary service selected the villages with the highest pig population. This might caused a bias, as, contrary to the Lao PDR, most of the pig farmers interviewed in Cambodia were raising pigs for commercial purposes only and keeping them in pens on a full-time basis. Extrapolating the outcomes of this survey in Cambodia might therefore not be possible.

The survey confirms that biosecurity implementation remains inadequate among smallholder pig producers in both countries, with average implementation rates of 43% in Cambodia and 27% in the Lao PDR. These results are consistent with observations across Southeast Asia, where backyard and semi-commercial farms—characterized by poor infrastructure, inadequate waste disposal, and high-risk practices such as swill feeding—have significantly contributed to the rapid spread of ASF in previously unaffected areas [9]. As a vaccine was developed and started being commercialized in some Asian countries, the future usage of that vaccine in the Lao PDR might help control the disease in the future. Nevertheless, it appears that, if close to 50% of the farmers (45% in Cambodia and 47% in The Lao PDR) believe that vaccination protects their herd against ASF (Table 4), only 52% of the Cambodian farmers and 42% of the Lao PDR vaccinated their pigs over the last 12 months (vaccine against the classical swine fever). If an ASF vaccine were to be commercialized in these countries, an intensive training and awareness campaign should therefore be organized to ensure proper coverage of the pig population within the country.

With respect to ASF awareness and knowledge, although most farmers had heard of the disease, 24% doubted its presence in their country (15% in Cambodia and 30% in the Lao PDR). This is unexpected, considering that ASF outbreaks have been reported since May 2019, with severe impacts and widespread awareness campaigns. The scepticism may reflect the lack of laboratory confirmation for many outbreaks and farmers’ limited ability to recognize ASF symptoms. Only 57% of respondents reported confidence in identifying ASF, and the mean knowledge score was only 28/100. According to case investigations from early ASF outbreaks in the Lao PDR, the most frequently reported early clinical signs were “anorexia (25.9%), weakness (29.9%) and sudden death (32.0%)”. Late-stage signs included “skin spots or reddened body (6.8% and 15.3% respectively) and death (40.7%)” [30]. Most of these signs are nonspecific, except for reddened skin, which was only recognized as an ASF clinical sign by 61% of Cambodian and 44% of Lao farmers. Nevertheless, the observed correlation between farmers’ perceived capacity to recognize ASF clinical signs and their actual knowledge was positive.

In contrast, knowledge of ASF transmission pathways was poor, with an average score of 34/100, and farmers’ perceived understanding did not align with actual knowledge. Many transmission pathways were underestimated. In Cambodia, only 31% of farmers recognized the risk of direct contact with infected pigs, 27% acknowledged the risks of swill feeding, and 47% recognized the risks of fomites. In the Lao PDR, underestimation was particularly notable for visitors (27%) and swill feeding (46%). In both countries, 52% of farmers believed that ASF could be transmitted via airborne routes, a misconception that may explain low BSM adoption. If farmers consider ASF transmission as uncontrollable, they may perceive biosecurity as ineffective. This aligns with the theory of planned behaviour, which emphasizes perceived behavioural control as one of the three determinants of compliance [17]. The perceived lack of control on possible ASF contamination linked to the overestimation of the risk of airborne transmission could therefore be an important barrier to BSMs implementation.

Overall, biosecurity implementation was low, with scores ranging from 7% to 79% (mean 43%) in Cambodia and 11% to 82% (mean 27%) in the Lao PDR. The sensitivity analysis identified three parameters that contributed to the biosecurity score at the farm level, in decreasing order: the type of pen walls (infrastructure), the implementation of 26 BSM in the farm (procedures), and the penning system with free ranging more at risk (management practice). In addition, several high-risk practices were observed, confirming earlier findings on ASF risk factors in Southeast Asia [9,29,31]. Free ranging was minimal in Cambodia (9%), likely because most respondents kept pigs for commercial purposes and because village selection targeted higher concentrations of pig farmers. However, in the Lao PDR, 32% of farmers practiced free ranging, consistent with previous findings [29,31]. Swill feeding was reported by 35% of respondents. Previous studies in the Lao PDR also noted that farmers often fed dogs with meat from ASF-affected pigs, with roaming dogs potentially serving as vectors of transmission [28,30]. The use of shared boars for breeding was widespread—72% in Cambodia and 24% in the Lao PDR—representing another major risk [32]. Non-formal and unregulated marketing pathways also present significant risks, as middlemen frequently move pigs between villages and markets. Despite this, 65% of farmers did not recognize visitors and their vehicles as a transmission risk, and 38% allowed unrestricted visitor access without any hygiene precautions.

Behavioural change is a progressive process influenced by several mental constructs such as farmers’ perceptions of disease risk, BSM’s benefits and barriers. The classification tree analysis identified three key mental constructs (ASF knowledge, risk perception, and perceived benefits of BSM) as major determinants of the biosecurity scores. Farmers with larger herds or greater commercial dependence were also more likely to perceive risks, recognize benefits, and achieve higher biosecurity scores. Education emerged as another determinant, exerting both direct effects on biosecurity and indirect effects through improved knowledge and risk perception.

Nevertheless, economic barriers remain significant for smallholders. The costs of pen construction, maintenance, and feed (as a substitute for scavenging) pose major challenges. Traditional practices and land tenure systems may also limit the adoption of BSM [33]. Practical, context-specific interventions that are low-cost, co-developed with farmers, and perceived as feasible and cost-effective are more likely to be adopted by smallholders and merit further investigations. As an example, a community based approach to biosecurity was tested in two villages of Toomlarn where the community decided to better prevent and control the disease at community level by prohibiting free ranging and mitigating the risks related to the middlemen, either by building a loading area and forbidding the access to the traders or by establishing a disinfection station for them to use before entering the village (unpublished data from AVSF).

## 5. Conclusions

This study provides the first large-scale comparative KAP survey on ASF in Cambodia and the Lao PDR, offering valuable insights into farmers’ perceptions and practices in diverse production contexts. Biosecurity implementation remains suboptimal, constrained by economic, cultural, and structural barriers, while high-risk practices continue to facilitate ASF spread. Despite widespread awareness of ASF, misconceptions about its transmission and persistence, combined with low knowledge scores, undermine effective disease prevention. Importantly, farmers’ education, herd size, knowledge, and perceptions of risk and benefits emerged as significant predictors of biosecurity adoption, underscoring the central role of behavioural and socioeconomic factors in disease control. Addressing these challenges requires tailored communication strategies that not only improve knowledge but also enhance farmers’ perception of control and the feasibility of BSM. Practical, affordable, and co-designed interventions are essential to ensure uptake by smallholders. Strengthening local veterinary services, promoting participatory extension, and integrating ASF control into broader livelihood and food security strategies will be critical to mitigating ASF’s impacts and enhancing the resilience of smallholder pig production systems in the region.

## Figures and Tables

**Table 1:** Scoring of the risk perceptions by the farmers.

Risk Perception	=(RP1 × RP2 + RP3 × R4 × RP5 + RP6)/10 × 100 (10 Being the Maximum of Points Possible)
RP1—Have you ever experienced an ASF outbreak on your farm?	0: No/1: Yes
RP2—Did any of your pigs die from ASF (after being sick or culled by local authorities) during the outbreak?	1: No/2: Yes
RP3—Do you know anybody who has been affected by ASF?	0: No/1: Yes
RP4—If yes, who?	1: Another pig farmer 1.5: A relative 2: A friend
RP5—If yes, where are these persons?	1: in another village 2: in the same village
RP6—How strongly do you agree with the following statement? (score from 1 to 4)	(Sum RP6.1 to RP6.4)/4
RP6.1—ASF is a very important disease	1 strongly disagree to 4 strongly agree
RP6.2—ASF is frequent in the country; if I do not take any measures, I will have an outbreak in my farm	2 strongly disagree to 4 strongly agree
RP6.3—My herd is not protected by vaccines and deworming	3 strongly disagree to 4 strongly agree
RP6.4—ASF is present in the Lao PDR	4 strongly disagree to 4 strongly agree

**Table 2:** Farmers’ characteristics.

	Cambodia (N = 188)	The Lao PDR (N = 283)	Overall (N = 471)
Percentage of women	68%	47%	56%
Age range	18–80	22–73	18–80
Education level			
Illiterate	19	45	64
Primary school	93	118	211
Secondary school	55	85	140
Higher education	21	35	56
African Swine Fever exposure			
Heard of ASF	92%	66%	77%
Experienced ASF	63%	52%	56%

**Table 3:** Farm characteristics.

	Cambodia (N = 188)	The Lao PDR (N = 283)	Overall (N = 471)
Herd size	1–110	1–1200	1–1200
Age range	18–80	22–73	18–80
Pig importance			
Main source of income	22%	30%	27%
Second source of income	50%	27%	36%
Third source of income	21%	17%	19%
Additional/not a source of income	6%	27%	14%
Years of pig farming			
<1 year	6%	20%	15%
>10 years	17%	24%	21%
>2–5 years	17%	12%	14%
>5–10 years	60%	43%	50%

**Table 4:** Risk perception.

	Cambodia (N = 188)	The Lao PDR (N = 283)	Overall (N = 471)
ASF is an important disease	73%	70%	71%
ASF is not present in Lao/Cambodia	15%	30%	24%
ASF is frequent; I need to take measures to prevent an outbreak on my farm	92%	66%	76%
My herd is not protected by vaccination and deworming	55%	53%	54%

**Table 5:** High-risk biosecurity practices.

Practice	Cambodia (N = 188)	The Lao PDR (N = 283)	Overall (N = 471)
Free ranging (full or partial time)	9%	48%	32%
Scavenging/feeding swill food	23%	43%	35%
Not reporting outbreak suspicions	66%	70%	69%
Selling/eating, or giving away sick pigs	31%	7%	17%
Not applying a two-week quarantine to purchased pigs	85%	61%	70%
Not isolating sick pigs	35%	38%	37%
Allowing visitors	44%	34%	38%
Throwing/selling or eating carcasses	44%	6%	21%
Sharing boars for mating	72%	24%	43%
Total	188	283	471

**Table 6:** Classification tree analysis, the difference between two groups in the Lao PDR (practice score below and over 45).

Explanatory Variable	Variable Importance (Scale from 0 to 100)
Education	100
Perceived benefits of biosecurity measures	99.74
Herd size	83.54
Risk perception	80.31
The main purpose of pig farming	64
Importance of pigs for livelihoods	61.78
Knowledge of African swine fever	60.21
Age of the farmer	40.2
Type of pigs	38.73
Gender	0.47

**Table 7:** Importance of the different explanatory variables over the biosecurity score, the knowledge score, the risk perception, and the perceived benefits.

**(A) Importance of the three mental constructs on the biosecurity scores**								
**Explanatory Variable**			**Lao PDR** **Importance over the BS Score** **(Scale from 0 to 100)**			**Cambodia** **Importance over the BS Score** **(Scale from 0 to 100)**		
Perceived benefits of biosecurity measures			100			73.43		
Risk perception			82.26			24.85		
Knowledge of African Swine Fever			72.82			12.14		
**(B) Importance of the farm and farmers profiles on the biosecurity score and the mental constructs**								
	**Lao PDR**				**Cambodia**			
	**Importance over (Scale from 0 to 100)**				**Importance over (Scale from 0 to 100)**			
**Explanatory variable**	**BS Score**	**Knowledge**	**Risk** **Perception**	**Benefits Perception**	**BS Score**	**Knowledge**	**Risk** **Perception**	**Benefits** **Perception**
Education	91.64	97.26	41.35	55.62		57.68	100	
Herd size	54.93		100	46.73	100	67.68	1.67	63.33
Importance of pigs for livelihoods	39.42		46.89	97.77	1.31	15.14	79.58	100
Main purpose of pig farming	29.97		4.83	100			61	100
Type of pigs	27.41		57.51	67.64		100	61.76	
Age of the farmer	17	100	1.88		1	0.01		
Gender	2.43					63.96		

## Data Availability

The data that support the findings of this study are available from the corresponding author upon reasonable request.

## Appendix A

**Table A1:** Scoring system.

Code	Variable	Score
**KS**	**Knowledge score**	**=(K1 + K2 + K3)/3 × 100**
K1	Have you ever heard about ASF?	0: No
		1: Yes
K2	Which of the following clinical signs do you associate with ASF in pigs?	Sum divided by 19 (maximum amount of points possible)
K2.1	Fever	0: symptom not selected
K2.2	Sudden death	2: symptom selected
K2.3	Presence of red loose skin coloration in the ventral abdomen, tips of ears, tail, or distal limb	
K2.4	Blood in diarrhea/eyes/nose/urine	
K2.5	Diarrhea	0: symptom not selected
K2.6	Higher mortality	1: symptom selected
K2.7	Joint swelling	
K2.8	Coughing/dyspnoea	
K2.9	Vomiting	
K2.10	Loss appetite	
K2.11	Abortion	
K2.12	Increase in water intake and wallowing	
K2.13	Seizures	
K2.14	Swelling face/eyes	
K2.15	Weakness	
K3	By which of the following spread pathways can pigs be infected with ASF?	Sum of points divided by 9 (total amount of points possible)
K3.1	Direct contact with an infected pig	0–1 (if not selected/if selected)
K3.2	Contact with pork product/carcass with contamination	0–1 (if not selected/if selected)
K3.3	Feeding of infected pig meat/swill/offal to pigs	0–1 (if not selected/if selected)
K3.4	Contact with infected wild boars	0–1 (if not selected/if selected)
K3.5	Visitors spreading the germs (e.g., pig traders)	0–1 (if not selected/if selected)
K3.6	Other domestic animals	0–1 (if not selected/if selected)
K3.7	Vehicles or equipment spreading the germs	0–1 (if not selected/if selected)
K3.8	Biting insects (ticks, fleas…)	0–1 (if not selected/if selected)
K3.9	Through the wind/air	1–0 (if not selected/if selected)
K3.10	None of the above	0 if selected
**RP**	**Risk perception score**	**0**
		(10 being the maximum number of points possible)
RP1	3.2 Have you ever experienced an African swine fever outbreak on your farm?	0: No/1: Yes
RP2	3.7 Did any of your pigs die from ASF (after being sick or killed by local authorities) during the outbreak?	1: No/2: Yes
RP3	3.6 Do you know anybody who has been affected by ASF?	0: No/1: Yes
RP4	Who do you know who has been affected by ASF?	1: Another pig farmer/1.5: A relative/2: A friend
RP5	Where these persons affected by ASF are keeping the pigs:	1: in another village/2: in the same village
RP6	4.1 How strongly do you agree with the following statement?	(SumR P6.1 to RP6.4)/4
RP6.1	ASF is a very important disease	Score from 1 (do not agree at all) to 4 (fully agree)
RP6.2	ASF is frequent in the country; if I do not take any measures, I will have an outbreak on my farm	
RP6.3	My herd is not protected by vaccines and deworming	
RP6.4	ASF is present in the Lao PDR	
**PI**	**Perceived importance measures**	**=Sum (A2.1 to A2.25)/25 × 100**
		(25 being the maximum number of points possible)
	Which following measures regarding ASF prevention and control do you consider efficient/important?	
PI.1	Having a foot bath at the entrance	0: Not selected/1: selected
PI.2	Purchasing a new pig, keeping it in quarantine for at least 2 weeks before mixing it with the others	0: Not selected/1: selected
PI.3	Isolating sick pigs from the others	0: Not selected/1: selected
PI.4	Not allowing visitors (e.g., butcher/middlemen/relatives…) to enter the pig pen	0: Not selected/1: selected
PI.5	Asking visitors entering the farm/the pens to change their shoes	0: Not selected/1: selected
PI.6	Asking visitors entering the farm/the pens to change clothes	0: Not selected/1: selected
PI.7	Asking visitors entering the farm/the pens to disinfect their shoes	0: Not selected/1: selected
PI.8	Not visiting other pig farms frequently (>once/week)	0: Not selected/1: selected
PI.9	Protecting the pigs’ feed from possible contamination by wildlife (Stored in a closed place)	0: Not selected/1: selected
PI.10	Keeping the pigs’ pens clean and dry all the time	0: Not selected/1: selected
PI.11	Not feeding pigs with swill food	0: Not selected/1: selected
PI.12	Vaccinating the pigs every 6 months	0: Not selected/1: selected
PI.13	When purchasing pigs, ask if there is an ongoing outbreak in the community or farm from where you are buying the pig	0: Not selected/1: selected
PI.14	Keeping piglets, sows, and boars in separate pens	0: Not selected/1: selected
PI.15	Having a draining system	0: Not selected/1: selected
PI.16	Using specific tools (not used for other animals) to take care of the pigs (e.g., shovels…)	0: Not selected/1: selected
PI.17	Using specific tools for each of the pig pens (e.g., shovels…)	0: Not selected/1: selected
PI.18	Using specific clothes/footwear for taking care of pigs (Different from your daily life clothes/footwear)	0: Not selected/1: selected
PI.19	Using manure to fertilize crops	0: Not selected/1: selected
PI.20	Not sharing boars between pig farms (lending or borrowing)	0: Not selected/1: selected
PI.21	Using all replacement stocks that are produced and grown within your farm/not buying pigs from outside	0: Not selected/1: selected
PI.22	Disinfection pig pen	0: Not selected/1: selected
PI.23	Mosquito net/spray	0: Not selected/1: selected
PI.24	Personal hygiene	0: Not selected/1: selected
PI.25	Use artificial intelligence	0: Not selected/1: selected
**BSc**	**Biosecurity score (BSc)**	**=(P1 × P1.1 × P1.2 + P2 + P3.1 + P4 + P5 + P6)/41 × 100**
**P1**	**Penning**	0: No, Full-time free-ranging/scavenging
		1: Part-time housed/fenced/penning
		2: Yes, full-time housed/fenced/penning
P1.1	Pen flooring	0: soil
		1: wooden floor
		2: cemented/concrete floor
P1.2	Pen walls	0: not precise/none
		1: wooden fences
		2: steel fences
		3: Solid walls
**P2**	**What are you feeding your pigs with now?**	0: scavenging/swill food
		1: Local feed
		2: Commercial feed
		Remark: if several, the one with the lowest score was attributed
**P3**	**If you observe clinical signs of ASF in your pig herd, what do you do? Action**	0: I would sell/eat/give the pigs as soon as possible to avoid losing too much
		1: I would treat the pigs/I would wait a few days to see if the pigs improve or not
		2: I would take additional BS measures /I would isolate the pig/I would bury/burn the carcass
**P4**	**If you suspect there is an ASF outbreak in your farm/village, what would you do:**	0: Nothing/Wait a few days before reporting it to avoid a false report/have the time to sell the healthy pigs and avoid too much losses/self treatment/vaccination
		1: Report it as soon as possible, even if it might be a false case
		2: Protect my farm by additional BS
**P5**	**Carcass disposal (pigs that died and are not consumed)**	=(P5.1 + P5.2)/3
P5.1	Do you have a carcass disposal point (CDP)?	0: No/1: Yes
P5.2	How do you dispose of carcasses?	0: Throw it into the bush/Sell it off/No dead pigs
		1: Burning/Burying
		2: Burying + Use of chemical
**P6**	**Which of the following practices are you implementing?**	=SOMME (P6.1 to 6.2&6.21)
P6.1	1. Do you have a foot bath at the entrance of your pens	0: No/Yes
P6.2	2. The last time you purchased a new pig, did you keep it in quarantine for at least 2 weeks before mixing them with the others?	1: Yes/No
P6.3	3. The last time one of your animals was sick, did you isolate it from the others?	N/A: not applicable
P6.4	4. Do you allow visitors (e.g., butcher/middlemen/relatives…) to enter the pig pen?	
P6.5	5. Do you ask visitors entering the farm/the pens to change footwear?	
P6.6	6. Do you ask visitors entering the farm/the pens to change clothes?	
P6.7	7. Do you ask visitors entering the farm/the pens to disinfect their shoes?	
P6.8	8. Do you visit other pig farms frequently (>once/week)	
P6.9	9. Do you protect the pigs’ feed from possible contamination by wildlife? (Stored in a closed place)	
P6.10	10. Do you keep the pigs’ pens clean and dry all the time?	
P6.11	11. Do you ever feed your pigs with swill food?	
P6.12	12. Did you vaccinate your pigs over the last 12 months?	
P6.13	13. The last time you purchased pigs, did you ask if there was an ongoing outbreak in the community or farm from where you are buying the pigs?	
P6.14	14. Are the piglets, sows, and boars kept in separate pens?	
P6.15	15. Do you use a drainage system?	
P6.16	16. Do you use specific tools when taking care of your pigs (e.g., shovels…)?	
	Meaning tools that you do not use for other animals	
P6.17	17. Do you use specific tools only for each pig pen (e.g., shovels…)?	
P6.18	18. Do you wear specific clothes/footwear for taking care of pigs?	
	(Different from your daily life clothes/footwear)	
P6.19	19. Do you use pig manure for fertilizing crops?	
P6.20	20. Do you share boars with other farms (lend out or borrow)?	
P6.21	21. Are all replacement stocks produced and grown within your farm?	
P6.22	5.8 Is there any other measure you are taking to prevent or control diseases that have not been listed?	0: No or measure not relevant/1: Yes and relevant
